# Exploring the factors influencing the adoption of online medical services by older adults: a modified UTAUT2 based study

**DOI:** 10.3389/fpubh.2025.1559701

**Published:** 2025-04-03

**Authors:** He Huang, Jiayi Zhu, Juhua Chen, Yuxin Qin, Shuguang Chen

**Affiliations:** ^1^College of Art and Design, Changzhou University, Changzhou, China; ^2^Department of Encephalopathy, Changzhou Traditional Chinese Medicine Hospital, Changzhou, China

**Keywords:** online medical services, older adults, UTAUT2, technology acceptance, structural equation modeling

## Abstract

**Objective:**

The adoption rate of online medical services (OMS) among older adults remains notably low. Existing literature on OMS has rarely focused on older adults and the influences of individual characteristics within this population remain underexplored. To explore the factors influencing the adoption of OMS by older adults in China, this study proposes a modified Unified Theory of Acceptance and Use of Technology 2 (UTAUT2) model by integrating technology anxiety, trust, and perceived risk and adding new moderating variables such as education level and health status.

**Method:**

Data was collected through a face-to-face survey, which included 379 valid questionnaires. Structural equation modeling (SEM) was used to analyze the data and test the research hypotheses.

**Results:**

For older adults, performance expectancy (*β* = 0.375, *p* < 0.001), effort expectancy (*β* = 0.244, *p* < 0.01), social influence (*β* = 0.198, *p* < 0.01), and trust (*β* = 0.237, *p* < 0.01) positively influence the usage intention of OMS, whereas technological anxiety (*β* = −0.129, *p* < 0.01) and perceived risk (*β* = −0.296, *p* < 0.001) negatively influenced the usage intention of OMS. No significant influence of facilitating conditions (*β* = 0.057, *p* = 0.293) or price value (*β* = 0.035, *p* = 0.721) on usage intention was found in this study. Meanwhile, the age, education level, and health status of the participants were found to moderate the effects of some major constructs on usage intention significantly.

**Discussion:**

Our empirical research discovers the drivers and barriers influencing the adoption of OMS by older adults. Based on the findings, we provide some recommendations to promote the adoption of OMS among older adults. Our findings and recommendations can aid providers, developers, policymakers, market practitioners, and managers of OMS in developing better services and strategies to successfully implement OMS among the older population.

## 1 Introduction

As an important aspect of the Internet integrating into people’s daily lives, the combination of Internet technology and medical services has given birth to online medical services (OMS), which have developed rapidly in the past decade. The rapid update of communication technology such as 5G and the isolation from offline medical services caused by the COVID-19 pandemic have further accelerated the development of OMS (1, 2). At present, OMS have become very important healthcare means coexisting with offline medical services. Compared with the latter, OMS transcends the limitation of time and space. It not only avoids the time consumption of traveling and queuing, thus making medical care more convenient, but also enables patients to more easily access high-quality and freely selectable medical resources by transcending geographical limitations (3, 4).

According to the report from the China Internet Network Information Center (CNNIC), the number of internet users in China has exceeded 1.072 billion by 2023, with 33.8% (approximately 364 million) of internet users having used relevant OMS (including appointment registration, via text and image consultation, via telephone/video consultation, report inquiry, online prescribing, drug purchase, etc.). However, the proportion of the users of OMS among older adults (aged 60 years and above) is only 12.83% (5), and most older adults are still using traditional face-to-face offline methods to obtain medical services. Although there are many benefits of OMS aforementioned, the low adoption rate of OMS among the older population represents that most of older adults have not benefited from OMS. Therefore, researching and promoting the adoption of OMS for older adults has become an important and meaningful theme, aimed at addressing the digital divide and achieving an inclusive society (6, 7).

Although OMS are relatively new things, a considerable amount of research has been carried out on their adoption and acceptance. Previous studies on OMS were conducted from different perspectives. Some of them explored the acceptance of OMS by the users (patients) (4, 8, 9), and some others analyzed the adoption of OMS by physicians (10). Recently, there was also cross-cultural research on the adoption of OMS (11), and even the business model of OMS has also been explored (12). Given the uneven distribution of medical resources in most developing countries, OMS have received more attention for their accessibility beyond time and space limitations (13–16). Previous studies used questionnaires or online surveys to investigate the factors facilitating or inhibiting the adoption of OMS by patients or physicians and to understand the underlying mechanisms, to provide policymakers, governments, software developers, medical institutions, insurance companies, and other stakeholders with recommendations on the design and promotion of OMS (11, 14, 17).

However, previous studies rarely involve or focus on older adults, or the proportion of older adults among their participants was very low. Due to physiological characteristics such as multiple illnesses, older adults need to use medical services more frequently compared with the general population (18). Moreover, the degradation of mobility also makes it difficult for a considerable proportion of older adults to make a trip (such as traveling to the hospital). Thus, OMS should have greater practical value for older adults, which seemed to be overlooked in previous studies.

Furthermore, as a special type of technological service, OMS is closely linked to the physiological health and disease conditions of users. It also requires users to possess both Internet technology experience and health knowledge (19), so the differences in demands and willingness to use OMS among older adults with different health statuses and education levels should be taken into account. However, existing literature has rarely explored the differences in these individual characteristics. Therefore, more in-depth insights into the heterogeneity issues of this age population are required for the research on the adoption of OMS by older adults, to provide more detailed references for promoting the adoption of OMS among older adults.

Hence, to fill these two gaps, this study explored the factors influencing the adoption of OMS by older adults through questionnaires and analysis of the proposed modified Unified Theory of Acceptance and Use of Technology 2 (UTAUT2) model. The purposes of this study was (1) to investigate the drivers and barriers influencing the adoption of OMS from the perspective of older consumers; (2) to explore the differences in the adoption of OMS among older adults attributed to individual characteristic variations; (3) to derive practical insights to promote the adoption of OMS among older adults based on the analysis of the influencing factors.

## 2 Theoretical basis

Technology acceptance has been a research topic for a long time and has been ongoing to today. In recent decades, new technological objects have been developed more rapidly and have been much more abundant in both quantity and category. People’s intention to use new products or technologies becomes crucial for the successful implication of the new technological objects, so technology acceptance research focuses on exploring the factors affecting the usage intention and underlying mechanisms. Overall, current research on technology acceptance of older adults is mostly focused on information and communication technology (ICT) based products and services, such as smartphones, touchscreen devices, smart wearable systems, mobile payments, mobile health, etc. Some representative models in the field of technology acceptance have also been used by researchers, such as the Technology Acceptance Model (TAM), Theory of Planned Behavior (TPB), UTAUT, and UTAUT2 (20–24). Chen and Chan (25) proposed a senior technology acceptance model (STAM) in 2014, which explored the factors influencing the adoption of a wide range of gerontechnology by older adults in general. The authors also acknowledged the need to adjust the model according to the context of different types of technologies, to provide reference on the development, design, and policy-making in a specific technological field.

Among these models, UTAUT2 is a recent and relatively more comprehensive model that has been proven to be widely effective in different application fields. It not only has a higher percentage of variance in usage intention explained than other models (26), but more importantly, based on the four important variables in its initial version (UTAUT), namely performance expectation, effort expectancy, social influence, and facilitating conditions, the addition of three new variables (hedonic motivation, price value, and habit) makes it more suitable for application in the consumer setting (27). In addition, it also includes three moderating variables: age, gender, and experience, to reflect the influence of differences in individual characteristics.

Although UTAUT2 has been widely applied in general technology acceptance research, its utilization remains limited in studies specifically targeting older adults. Macedo (20) analyzed the acceptance and use of ICT by older adults by using UTAUT2. Her research included a wide range of online activities (such as sending/receiving emails, shopping online, browsing news, seeking information on health, etc.) and it was a general study that just totally copied the UTAUT2 model. Xu et al. (28) combined performance expectancy, social influence, and hedonic motivation with perceived health threat, threat appraisal, and health literacy to analyze the intention of older adults to continuously use the maintenance-oriented WeChat official accounts. A more recent study integrated UTAUT2 with the Technology Readiness Index model explored the factors influencing the use of smart wearable devices by older adults (29).

Moreover, to the best of our knowledge and literature review, UTAUT2 remains practically nonexistent in investigations of older adults’ OMS acceptance context. Given that OMS is an emerging object which combining Integrates internet communication technology and professional medical services, its unique characteristics and impacts necessitate careful consideration of applying UTAUT2 to analyze older adults’ adoption of this service. Firstly, OMS is a relatively new technological service with a low penetration rate among the older population (The majority of them belong to non-users), which is worth noting. Older adults are more likely to experience anxiety or fear towards new technologies that they have not used before (30), which would have a significant influence on their usage intention (25). Therefore, it is necessary to incorporate technology anxiety into the acceptance model for analysis. Secondly, OMS is in a special service context with strong professionalism (different from daily browsing news, shopping online, chatting, and other online activities), so factors such as users’ trust in the reliability of the technological service, and concerns about the risks of their privacy and personal information should also be considered in the research. In addition, OMS are important components of the healthcare field. Previous studies also found that different health conditions of older adults could affect the adoption of healthcare technologies (22, 25). Therefore, it is necessary to include the health status of older adults as an important individual factor in the research model.

## 3 Research model and hypotheses

**Figure 1:**
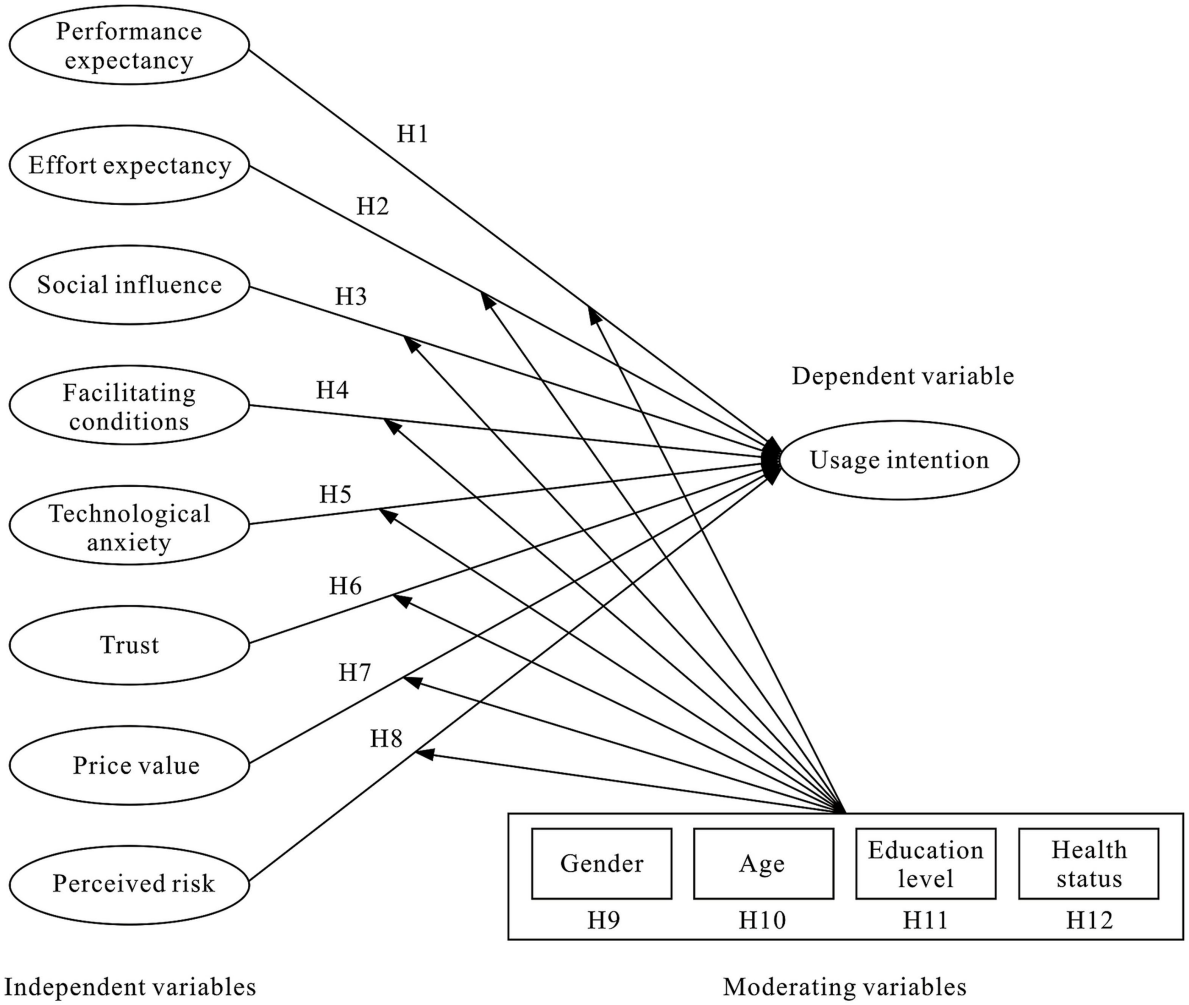
Proposed model of this study.

In this study, we made modifications to the UTAUT2 model as follows: Firstly, given it was difficult to associate the purpose and experience with fun, cheerfulness, enjoyment, and entertainment in the context of medical consultation. Although a recent study on OMS included hedonic motivation in its modified UTAUT2 model, the authors also acknowledged that using a medical consultation was never a pleasure (11). Therefore, we dropped hedonic motivation from this study. Secondly, in UTAUT2, habit and experience were two related yet distinct constructs. Venkatesh et al. (27) argued that experience was a necessary condition for forming habits, and differences in habits were caused by different usage times and levels of familiarity, all of these were related to the actual usage time and experience of a technology. Due to the low adoption rate of OMS among older adults aforementioned, which means most of older adults were non-users of OMS with no previous usage experience. As a result, habit and experience were dropped from this study. Correspondingly, for the same reason, usage behavior in UTAUT2 was also dropped, and usage intention (UI) was the sole dependent variable. Lastly, given the particularity and professionalism in the technological service context of OMS aforementioned, we introduced technical anxiety, trust, and perceived risk, and added the health status and education level of the older adults as moderator variables. Therefore, there were nine main constructs namely performance expectancy, effort expectancy, social influence, facilitating conditions, technological anxiety, trust, price value, and perceived risk, and four moderating variables namely age, gender, education level, and health status in the modified UTAUT2 model. Figure 1 presents the proposed model of this study. The following sections discussed the variables in our modified UTAUT 2 model and the corresponding hypotheses.

### 3.1 Performance expectancy (PE)

Performance expectancy is typically defined as the degree to which an individual believes that using a technology can help improve their job performance (31). PE originates from “perceived usefulness” in the TAM model (32), which mainly reflects the practical use and benefits brought by using new technology. People are more willing to use a new or special technology when they believe that it can improve their work performance and life quality compared with traditional approaches (31, 33, 34). In this study, PE refers to the usefulness and convenience that older adults believe using OMS could help them access medical services and consultation outcomes better. Previous studies have also found that older adults are more inclined to accept a new technology when they believe it can bring more benefits and make their lives easier (30, 35). Meanwhile, in the field of healthcare, existing literature has also found that PE is a very important predictor of usage intention (4, 11). Therefore, the first hypothesis of this study is as follows:

*H1:* Performance expectancy positively influences the usage intention of OMS by older adults.

### 3.2 Effort expectancy(EE)

Effort Expectancy is defined as “the degree of ease associated with the use of the system” (31). EE is adapted from the “perceived ease of use” in the TAM model (32), which is usually used to measure an individual’s perceived effort in using or learning to use a certain technology. In the research context of this study, EE reflects the degree to which older adults find OMS easy to use. Previous studies on technology acceptance by older adults have acknowledged that effort expectancy is a significantly positive predictor of an individual’s intention to utilize technology (20, 36). In the acceptance of new technology, especially in the initial use of the technology or acceptance of its innovation, effort expectancy has a strong impact on an individual’s behavior intention (30). As OMS is not yet used by most older adults, this study estimates that if older adults feel that OMS is easier to learn and use, they will be more willing to adopt it as their way of accessing medical services. Therefore, the second hypothesis of this study states that:

*H2:* Effort expectancy positively influences the usage intention of OMS by older adults.

### 3.3 Social influence (SI)

According to Venkatesh et al. (31), Social influence is the degree to which an individual perceives others, especially his/her important acquaintances believe that he/she needs to use a certain technique. SI emphasizes the influence of the interpersonal environment and others’ opinions on an individual’s acceptance of technology. Some key interpersonal relationships such as family, friends, relatives, and neighbors can play an important role in the decision-making behavior of older adults (37). Macedo (20) found that SI positively predicted the behavioral intention of older adults to use ICTs. In the survey by Huang et al. (30) SI was also found to have a significant positive impact on the older adults’ intention to use gerontechnology. Although in terms of OMS, studies from young and middle-aged participants have drawn inconsistent conclusions regarding the significant influence of SI on usage intention (11, 14, 38), this study still adopts the contribution of previous research on the technology acceptance of older adults to consider the significant positive correlation between SI and UI. Therefore, the third hypothesis of this study is proposed as follows:

*H3:* Social influence positively influences the usage intention of OMS by older adults.

### 3.4 Facilitating conditions (FC)

Facilitating conditions refers to the degree to which an individual believes that there are sufficient technological infrastructures and favorable conditions to support their use of new technologies (31). In UTAUT2 (27) and STAM (25), FC was an important factor that affected both behavioral intention and usage behavior, and existing research on technology acceptance also found that facilitating conditions significantly positively affected older adults’ intention to use technology (20, 22). In the context of OMS, FC includes online medical platforms (such as websites and apps), service support measures (such as online help, tutorials, and after-sales services), network conditions (such as wireless networks, 5G communications), and personal Internet devices (such as personal computers, mobile phones, tablets) that are sufficient to support OMS (12). A cross-sectional study from West China also found a positive effect of facilitating conditions on patients’ willingness to use online hospitals (4). Given OMS is a rather new approach to obtaining medical service, older adults may require more adequate technical resources and knowledge support, especially at the beginning of the learning and usage. Therefore, the fourth hypothesis of this study says:

*H4:* Facilitating conditions positively influence the usage intention of OMS by older adults.

### 3.5 Technological anxiety (TA)

Technological anxiety is commonly described as an individual’s apprehension or fear when he/she is faced with the possibility of using a new technology (31). We introduce this concept into the modified UTAUT2 because older adults rely more on the existing patterns based on gained experience, coupled with their decreased learning ability and memory, making them resistant and fear to use new technology (18). As an aging-specific factor, a negative relationship was found between using new technology and TA for older adults in several previous studies (25, 36). In the OMS context, some studies also revealed that TA was a significant barrier for individuals to use OMS (14, 18). Therefore, the fifth hypothesis of this study states as follows:

*H5:* Technological anxiety negatively influences the usage intention of OMS by older adults.

### 3.6 Trust (Tru)

Trust has also been taken into consideration in some studies on technology acceptance and has been confirmed to be an antecedent determinant influencing the usage intention (14, 39). Previous studies have also found that trust is a primary factor in understanding older adults’ perception of new technologies and plays an important role in their adoption of digital services (40). In the context of OMS in this study, trust refers to the degree of confidence that older adults have in the authenticity and professionalism of the services provided by doctors through the online platform. Because doctors and patients sometimes can not see each other (such as via text chat) and doctors can not touch patients, older adults may feel that communication on the Internet is not as real as face-to-face consultation. Meanwhile, the possible wrong results of online diagnostics will make them more cautious when choosing medical services (41). Here, trust is conceptually equivalent to professionalism (16) and reliability (13) as mentioned in some previous research on healthcare services, mainly including trust in the identity and qualifications of doctors, faith in their abilities and diagnostic results. The higher older adults believe in the reliability and the diagnostic results of OMS, the higher their adoption of this new medical service method will be. Consequently, we proposed the sixth hypothesis as follows:

*H6:* Trust positively influences the usage intention of OMS by older adults.

### 3.7 Price value (PV)

Although Venkatesh et al. (31) added price value as a determinant of the intention to use technology from the perspective of consumers in UTAUT2, it was relatively rare in practical applications. A meta-analysis showed that 79% of studies using UTAUT2 did not include price value in their models and most of them did not provide a reason (42). In the field of healthcare, the role of price value has not been fully studied. Unlike some countries’ free healthcare systems, medical services require payment and sometimes are expensive in the majority of developing countries including China. As an alternative channel for offline medical services, price value can be a key factor in decision-making by comparing the cost of the different channels and deciding whether to purchase or use them (17, 43). A cross-sectional study in China even found that price value was the strongest predictor for acceptance of pediatric telemedicine services (44). Following the above, price value was defined as a consumer’s cognitive trade-off between the monetary cost and perceived benefits of using OMS. Therefore, the seventh hypothesis was proposed as follows:

*H7:* Price value positively influences the usage intention of OMS by older adults.

### 3.8 Perceived risk (PR)

OMS is not only a form of medical services, but also an online information transmission and consumption behavior. Many previous studies believed that perceived risk referred to the user’s potential concerns with the problem of privacy and security when they were consuming online, and perceived risk was identified as a key barrier to adopting online consumption and services (45–47). In the OMS context, users need to disclose personal identity information and health information during the consultation process, and also disclose personal financial information during the payment process. An empirical survey also found that perceived risk was a major barrier to patients’ acceptance of telemedicine services (14). Furthermore, perceived risk is more essential for older adults as their risk tolerance reduces, so information security becomes a major concern for them when adopting OMS (36). When older adults perceive a higher risk of OMS, they are more inclined to be conservative and not make adoption decisions or may switch to using offline medical services, which they are more familiar with. Therefore, the eighth hypothesis was formulated as follows:

*H8:* Perceived risk negatively influences the usage intention of OMS by older adults.

### 3.9 Moderating variables

In UTUT2, there are also three moderating variables namely gender, age, and experience. Venkatesh et al. (27) argued that differences in individual characteristics could affect their attitudes toward adopting the technology and use behavior. However, in most studies on UTAUT2, these variables are often used as control variables to reduce experimental bias, and only a few studies analyzed their moderating effects on the relationship between independent and dependent variables (11, 48). The older population is a heterogeneous group and diverse in their characteristics such as age, gender, education level, and health status, all of which may influence the relationship between the aforementioned independent variables (such as PE, EE, SI, etc.) and the dependent variable (UI). Therefore, it is necessary to explore the effects of these moderating variables on older adults’ acceptance of OMS further, which will be beneficial for developing more detailed strategies to promote their adoption of OMS.

Previous studies have confirmed the impact of age on technology acceptance, suggesting that the older an individual is, the more difficult it is for him/her to learn, remember, and operate new technologies, leading to a decrease in the adoption rate of new technologies (11, 49).

Gender is the most commonly studied variable in the research of technology acceptance, but mixed results are obtained. Some studies found that men were more adept at accepting and using new technologies, possibly due to their more proactive attitude toward challenges (25, 50), whereas there were no significant gender differences in user behavioral intentions in some other studies (20, 22).

In addition, as the participants in this study were non-users with no prior experience in using OMS, we dropped the experience variable. Meanwhile, previous studies have found that different education levels of older adults significantly affect their acceptance of ICT technology and individuals with higher education levels are more likely to adopt new technology (20, 23, 51).

Finally, previous studies have also found that the different individual health conditions of older adults have a significant impact on the adoption of healthcare technologies and services (22, 25). Therefore, we used gender, age, educational level, and health status as the moderating variables in this study, and proposed the ninth, tenth, eleventh, and twelfth hypotheses as follows:

*H9:* The relationships between the main constructs and usage intention were moderated by age.

*H10:* The relationships between the main constructs and usage intention were moderated by gender.

*H11:* The relationships between the main constructs and usage intention were moderated by education level.

*H12:* The relationships between the main constructs and usage intention were moderated by health status.

## 4 Materials and methods

### 4.1 Measurements

The survey questionnaire in this study consists of two parts. The first part was demographic variables such as gender, age, marital status, income, education level, and health status. Education level is divided into secondary school or below, high school, and college or above according to previous research (22). Health status was divided into three levels, namely healthy, basically healthy, and poor healthy. It was based on the assessment of physical health in the standard for healthy Chinese older adults released by the National Health Commission, which included three parts: general condition, daily living activity ability, and disease status (52). The second part consisted of the nine constructs included in this study. The items of each construct were adapted from previously validated and reliable scales to fit the OMS context. Most of the items in the questionnaire (including PE, EE, SI, FC, PV, and UI) were based on UTAUT2 (27), while the items for TA were adapted from Venkatesh et al. (31) and Chen and Chan (25). Three items measuring Tru were adapted from McCloskey (53) and Kamal et al. (14). The scale of PR was based on items used by Li et al. (22) and Kamal et al. (14).

All items on the scale ranged from 1 to 7 using a 7-point Likert scale, with 1 representing strongly disagree and 7 representing strongly agree. The initial questionnaire was written in English and then translated into Chinese. Language experts from the English major of the author’s university were invited to proofread the questionnaire to ensure the accuracy of the translation. A pilot study was conducted in which 30 older adults from the communities near the author’s university were recruited to fill out questionnaires and their feedback was obtained. At the same time, experts in the field of Internet applications and medical services were also invited to evaluate and assist in improving the effectiveness of the questionnaire. After several rounds of revision and review, the final questionnaire was formed. All the constructs and items of the final questionnaire are listed in Appendix A (See the Supplementary material).

### 4.2 Data collection

The data was collected through a face-to-face questionnaire-based survey. The minimum required sample size for the proposed model in this study was 290, which was determined by the rule of thumb that the sample size should be at least 10 times the number of items of the model (54). A total of 420 questionnaires were distributed and 379 valid questionnaires were collected, resulting in a completion rate of 90.24%. Participants were Chinese older adults aged 60 years or above recruited from six big communities/administrative villages using a convenience sampling method in Changzhou, China. Changzhou has the typical characteristics of cities in the eastern region of China, with a high degree of population aging, comprehensive medical resource allocation, and advanced digital infrastructure, which are conducive to the promotion and investigation of OMS among older adults.

Our trained interviewers conducted the survey with the assistance of the staff of the community/village committee. After obtaining the participants’ consent, the interviewers distributed the questionnaires and the participants filled them out on-site. During the survey process, for some participants who had difficulty in reading, the investigator would read the questions to the participants and fill in the answers on their behalf. At the same time, some images, user interfaces (such as apps), Demo videos, and charging standards (such as fees of the platform) of OMS were displayed to the participants to help them understand and evaluate OMS better. During the survey, participants could also express their opinions and suggestions on OMS. It took about an average of 40 min for the participants to complete the questionnaire.

The average age of the participants was 72.39 years, 43.8% of the participants were male and 56.2% were female. Nearly half of the participants were living with a spouse (47.8%). Regarding the education level, the maximum frequency of participants (39.8%) had undergone high school education. The main income of the participants was between 3,000 and 5,000 RMB (49.3%). Additionally, the self-rated health of the participants was also obtained from a questionnaire using the Chinese Healthy Elderly Assessment Scale (52) and it was found that the proportion of participants with basically healthy was the largest (44.6%). Besides, all the participants had no prior experience in using OMS. Table 1 shows the demographic characteristics of the participants.

**Table 1:** Demographic characteristics of the participants.

Characteristics	Frequency	Percentage (%)
Gender		
Male	166	43.8
Female	213	56.2
Age		
60–64	102	26.9
65–69	114	30.1
70–74	87	23.0
75 and above	76	20.1
Living condition		
Living alone	76	20.1
Living with spouse	181	47.8
Living with children	98	25.9
Others	24	6.3
Education level		
Secondary school or below	108	28.5
High school	151	39.8
College or above	120	31.7
Monthly income(RMB)		
Low (<3,000)	121	31.9
Middle (3,000 ~ 5,000)	187	49.3
High (> 5,000 RMB)	71	18.7
Health status		
Healthy	111	29.3
Basically healthy	169	44.6
Poor healthy	99	26.1

## 5 Results

### 5.1 Measurement model assessment

In this study, the data was analyzed using a two-step approach containing a measurement model and a structural model, which were recommended by Anderson and Gerbing (55). To assess the measurement model, it is necessary to evaluate its reliability and validity. Reliability refers to the internal consistency of the data and construct validity refers to the validity of the model including convergent validity and discriminative validity. Through Confirmatory Factor Analysis (CFA) using AMOS 24.0 software, the standardized factor loadings, cross-loadings, combination reliability (CR), average variance extraction (AVE), and Cronbach’s alpha coefficient (Cronbach’s alpha) of each variable were obtained. As CFA in AMOS can not directly obtain Cronbach’s alpha coefficient, SPSS 26.0 software was also used for calculating Cronbach’s alpha coefficient. The results showed that the CR values of all constructs in the model were higher than 0.7, and Cronbach’s alpha values were higher than 0.7, indicating that the proposed model had a high reliability (56). All items in the model had loadings higher than 0.7 in their corresponding factors, and the AVE values of all constructs were also higher than 0.5, indicating that the model had a good convergent validity (57), as shown in Table 2.

**Table 2:** The standardized factor loadings, cross-loadings, values of CR, AVE, and Cronbach’s alpha.

Constructs	Items	Factor loadings	Cross-loadings^a^	CR	AVE	Cronbach’s alpha
PE	PE1	0.857	0.212	0.932	0.821	0.853
	PE2	0.931	0.132			
	PE3	0.929	0.139			
EE	EE1	0.834	0.228	0.933	0.776	0.812
	EE2	0.876	0.137			
	EE3	0.881	0.204			
	EE4	0.840	0.195			
SI	SI1	0.885	0.185	0.945	0.851	0.907
	SI2	0.906	0.122			
	SI3	0.946	0.155			
FC	FC1	0.770	0.239	0.897	0.686	0.797
	FC2	0.892	0.182			
	FC3	0.852	0.169			
	FC4	0.793	0.249			
TA	TA1	0.935	0.145	0.927	0.807	0.783
	TA2	0.869	0.139			
	TA3	0.880	0.127			
Tru	Tru1	0.858	0.142	0.890	0.731	0.866
	Tru2	0.913	0.139			
	Tru3	0.791	0.144			
PV	PV1	0.920	0.166	0.927	0.805	0.759
	PV2	0.867	0.197			
	PV3	0.903	0.152			
PR	PR1	0.865	0.162	0.913	0.779	0.834
	PR2	0.963	0.140			
	PR3	0.814	0.147			
UI	UI1	0.941	0.119	0.917	0.787	0.822
	UI2	0.856	0.149			
	UI3	0.862	0.137			

^a^To simplify the table, only the max cross-loadings are shown. Full factor loadings can be found in Appendix B (See the Supplementary material).

Regarding discriminative validity, the results showed that the cross-loadings of all items were below 0.4 (Table 2 and Appendix B in the Supplementary material), and the square roots of AVE for all constructs exceeded the pairwise correlations between constructs, indicating discriminative validity was satisfied according to the Fornell-Larcker criterion (57, 58), as shown in Table 3.

**Table 3:** Correlation coefficient matrix and discriminant validity.

	PE	EE	SI	FC	TA	Tru	PV	PR	UI
PE	**0.906**								
EE	0.602	**0.881**							
SI	0.238	0.553	**0.923**						
FC	0.409	0.412	0.501	**0.828**					
TA	0.350	0.530	0.493	0.586	**0.899**				
Tru	0.436	0.450	0.473	0.231	0.269	**0.856**			
PV	0.252	0.449	0.642	0.506	0.312	0.453	**0.897**		
PR	0.409	0.376	0.283	0.325	0.373	0.527	0.351	**0.883**	
UI	0.276	0.619	0.524	0.472	0.611	0.336	0.219	0.307	**0.887**

PE, Performance expectancy; EE, Effort expectancy; SI, Social influence; FC, Facilitating conditions; TA, Technological anxiety; Tru, Trust; PV, Price value; PR, Perceived risk; UI, Usage intention. Boldface numbers on the diagonal represent the square root values of the Average Variance Extracted (AVE).

### 5.2 Structural model and hypotheses test

Before analyzing the structural model, it is necessary to first evaluate the fitness of the model, which is an important measure to test the predictive ability of the model. We used AMOS software to obtain the relevant fit indices of the model. As shown in Table 4, it can be seen that all indices have reached the corresponding criteria (56, 59, 60), which indicates that the proposed model has good fitness.

**Table 4:** Fitness of the model.

Indices	*χ*^**2**^/df	CFI	GFI	NFI	TLI	RMSEA	SRMR
Value	2.027	0.926	0.953	0.917	0.931	0.042	0.055
Criteria	<3	>0.9	>0.9	>0.9	>0.9	<0.05	<0.08

χ^**2**^/df: Ratio between chi-square and the degrees of freedom; CFI, Comparative fit index; GFI, Goodness of fit index; NFI, Normed fit index; TLI, Tucker-Lewis index; RMSEA, Root mean square error of approximation; SRMR, Standardized root mean squared residual.

In this study, structural equation modeling (SEM) was used to analyze the relationships among the constructs of the model. These relationships are mainly reflected through the path coefficients and their significance, which could be used to verify the hypotheses of this study. We performed calculations of the model using the Maximum Likelihood method in AMOS software, and finally obtained the results as shown in Table 5. Regarding the main constructs, the results showed that H1, H2, H3, H5, H6, and H8 were supported while H4 and H7 were not supported. That was, PE (*β* = 0.375, *p* < 0.001), EE (*β* = 0.244, *p* < 0.01), SI (*β* = 0.198, *p* < 0.01), and Tru (*β* = 0.237, *p* < 0.01) had a significant positive influence on usage intention, while TA (*β* = −0.129, *p* < 0.01) and PR (*β* = −0.296, *p* < 0.001) had a significant negative influence on usage intention. The influence of FC (*β* = 0.057, *p* = 0.293) and PV (*β* = 0.035, *p* = 0.721) on usage intention was not significant.

**Table 5:** Results of hypotheses testing.

Hypothesis		Path coefficients	Result
H1	Performance expectancy (PE) positively influences the usage intention of OMS for older adults.	0.375^***^	Supported
H2	Effort expectancy (EE) positively influences the usage intention of OMS for older adults.	0.244^**^	Supported
H3	Social influence (SI) positively influences the usage intention of OMS for older adults.	0.198^**^	Supported
H4	Facilitating conditions (FC) positively influences the usage intention of OMS for older adults.	0.057	Not Supported
H5	Technological anxiety (TA) negatively influences the usage intention of OMS for older adults.	−0.129^**^	Supported
H6	Trust (Tru) positively influences the usage intention of OMS for older adults.	0.237^**^	Supported
H7	Price value (PV) positively influences the usage intention of OMS for older adults.	0.035	Not Supported
H8	Perceived risk (PR) negatively influences the usage intention of OMS for older adults.	−0.296^***^	Supported
H9	The relationships between the main constructs and usage intention were moderated by age.	—	Supported
H10	The relationships between the main constructs and usage intention were moderated by gender.	—	Not Supported
H11	The relationships between the main constructs and usage intention were moderated by education level.	—	Supported
H12	The relationships between the main constructs and usage intention were moderated by health status.	—	Supported

^**^p < 0.01, ^***^p < 0.001.

Furthermore, the moderating effects of variables such as age, gender, education level, and health status were also examined, as shown in Table 6. Regarding age, the participants were divided into two groups: older adults aged 74 and below and older adults aged 75 and above, which was according to the international boundary of age distinguishing between young older adults and older adults. The results showed that significant age differences were found in EE (0.193 versus 0.297, *t* = 2.215, *p* < 0.01), FC (0.045 versus 0.112, *t* = 2.237, *p* < 0.01), and TA (−0.092 versus −0.185, *t* = −3.315, *p* < 0.001), with stronger effects for older adults aged 75 and above. Thus, H9 was supported. Regarding gender, he participants were divided into male and female groups, and no significant differences were found between these two groups in any construct, so H10 was not supported. In addition, regarding education level and health status, the participants were divided into three groups as aforementioned. The results showed significant differences in education level were found regarding EE (0.426 versus 0.193, and 0.426 versus 0.165, *t* = −2.633 and − 2.794, *p* < 0.001) and TA (−0.219 versus −0.106, and −0.219 versus −0.095, *t* = 1.985 and 2.173, *p* < 0.01), with stronger effects for older adults having education experience of secondary school or below. Thus, H11 was supported. Meanwhile, significant differences in health status were found regarding Tru (0.197 versus 0.553, and 0.237 versus 0.553, *t* = 3.259 and 2.763, *p* < 0.001) and PV (0.027 versus 0.126, and 0.051 versus 0.126, *t* = 2.308 and 2.091, *p* < 0.01), with stronger effects for older adults with poor healthy. Therefore, H12 was supported.

**Table 6:** The moderating effects of age, education level, and health status.

Path	Age			Gender			Education level						Health status					
	Estimate		Critical ratio	Estimate		Critical ratio	Estimate			Critical ratio ^a^	Critical ratio ^b^	Critical ratio ^c^	Estimate			Critical ratio ^d^	Critical ratio ^e^	Critical ratio ^f^
	60–74	≥75		Male	Female		Secondary school or below	High school	College or above				Healthy	Basically healthy	Poor healthy			
PE → UI	0.384^***^	0.323^***^	**−**0.528	0.396^***^	0.341^***^	**−**0.492	0.351^***^	0.392^***^	0.410^***^	1.053	1.324	0.979	0.353^***^	0.396^***^	0.417^***^	0.108	0.373	0.072
EE → UI	0.193^**^	0.297^***^	**2.215** ^**^	0.185^**^	0.257^**^	1.206	0.426^***^	0.193^**^	0.165^**^	**−2.633** ^***^	**−2.794** ^***^	**−**1.095	0.230^**^	0.252^**^	0.225^**^	0.694	**−**0.045	**−**1.203
SI → UI	0.187^**^	0.214^**^	0.039	0.176^**^	0.209^**^	0.245	0.232^**^	0.166^**^	0.203^**^	**−**1.226	**−**0.547	0.886	0.159^**^	0.225^**^	0.213^**^	1.569	1.257	**−**0.735
FC → UI	0.045	0.112^*^	**2.237** ^**^	0.049	0.072	0.119	0.067	0.051	0.039	**−**0.548	**−**0.952	**−**0.835	0.066	0.038	0.052	**−**1.485	**−**0.742	1.337
TA → UI	**−**0.092^*^	**−**0.185^**^	**−3.315** ^***^	**−**0.143^*^	**−**0.105^*^	0.261	**−**0.219^**^	**−**0.106^*^	**−**0.095^*^	**1.985** ^**^	**2.173** ^**^	0.698	**−**0.115^*^	**−**0.108^*^	**−**0.146^*^	0.069	**−**0.254	**−**0.392
Tru → UI	0.219^**^	0.242^**^	0.158	0.198^**^	**−**0.255^**^	**−**0.940	0.257^**^	0.211^**^	0.239^**^	**−**1.044	**−**0.865	0.593	0.197^**^	0.237^**^	0.553^***^	1.207	**3.259** ^***^	**2.763** ^***^
PV → UI	0.028	0.057	1.023	0.024	0.063	0.377	0.075	0.068	0.022	**−**0.259	**−**1.409	**−**1.027	0.027	0.051	0.126^*^	0.785	**2.308** ^**^	**2.091** ^**^
PR → UI	**−**0.279^***^	**−**0.315^***^	**−**1.487	**−**0.281^***^	**−**0.354^***^	**−**1.245	**−**0.323^***^	**−**0.291^***^	**−**0.277^***^	1.089	1.752	1.033	**−**0.254^**^	**−**0.323^***^	**−**0.309^***^	**−**0.334	**−**0.216	0.105

^a b c^ Referred to critical ratios for difference between Secondary school or below and High school, between Secondary school or below and College or above, and between High school and College or above, respectively; ^d e f^ Referred to critical ratios for difference between Healthy and Basically healthy, between Basically healthy and Poor healthy, and between Healthy and Poor healthy, respectively. Dark grids indicate the significant differences in comparison. ^*^p < 0.05, ^**^p < 0.01, ^***^p < 0.001.

## 6 Discussions

### 6.1 The influences of main constructs on the usage intention of OMS for older adults

This study used the modified UTAUT2 model to make it more suitable for the context of OMS for older adults. The influence of the main constructs on the usage intention of OMS by older adults was explored, to identify the drivers and barriers that affected their intention to adopt OMS.

First, the original three constructs PE, EE, and SI from UTAUT2 significantly positively affected the usage intention of OMS for older adults, which was consistent with the findings of previous research (11, 14, 20). PE has the highest coefficient path weight (*β* = 0.375, *p* < 0.001), which indicates it was the construct with the greatest impact on usage intention. This result suggested that older adults regarded the effectiveness and usefulness of technological services as the most important, and they were only willing to learn or use new technology when they perceived it useful (20, 30). The positive impact of EE on usage intention was found to exist among older adults in this study, which is in line with previous studies (36). It indicated that the ease of use of new technologies should be an important promoting factor for older adults with decreased learning ability and working memory (61). However, in a recent study by Schmitz et al. (11), no such impact of EE on UI was found among middle-aged and young adults. For them, there was a greater tolerance for the ease of operation of new technologies, and OMS would be just an expansion of Internet applications in their daily life, so there was not much difficulty involved. Therefore, the ease of use of new technological services is an important aspect of achieving their universal access, which deserves the attention of technology developers.

Similarly, SI had a significant positive impact on the UI of OMS among older adults, which was consistent with previous research on technology acceptance of older adults (20, 30). However, such results have not been found among middle-aged and young adults either (4, 11). Our results indicate that the external social environment has a significant impact on the adoption of new technologies by older adults, which may be because older Chinese adults tend to gather and socialize in groups, especially after retirement. They rely more on the information from word-of-mouth communication. Group conformity makes older adults trust their peers who are experiencing similar age-related changes in physical and psychological conditions more, and willing to communicate and share experiences (62).

Second, there were two constructs from UTAUT2, namely facilitating conditions (FC) and price value (PV), which did not have a significant influence on the usage intention of OMS for older adults. Regarding FC, our results were different from previous studies on the technology acceptance of older adults (20, 29), which was possibly due to the differences arising from the degree of infrastructure completeness and social culture. On one hand, the infrastructure required for OMS such as smartphones and computers had become popular in older adult households, and ubiquitous wireless networks also provided convenience. On the other hand, organizational support at a deeper level did not keep up. Although the Chinese government strengthened the promotion of OMS after the epidemic, it still focused on service providers such as hospitals, and publicity (63). The training and technical support for older adults (such as online assistance and customer service) were far from adequate, and most older adults still relied on their adult children to use OMS (30). Therefore, older adults did not perceive obviously that FC directly affected their usage intention of OMS.

Regarding PV, based on the best of our knowledge and literature review, this was the first time that price value has been included in the context of the acceptance of OMS. Surprisingly, the impact of PV on UI was not significant for older adults although OMS in China required payment and sometimes its fee was even higher than offline services (such as additional handling fees charged by the platform). The interpretation of this result might be: on the one hand, all the participants were non-users of OMS who lacked actual usage experience. Moreover, the transportation and time costs associated with offline medical services made the price comparison with OMS (which included handling fees) not obvious. On the other hand, Chinese older adults attach great importance to the value of health. They were even willing to spare no expense to obtain high-quality medical services (64), so they were not highly sensitive to the prices of OMS.

Finally, taking a closer look at the three new constructs integrated into the modified UTAUT2, this study found that technology anxiety (TA) had a significantly negative influence on the usage intention of OMS for older adults. Our result was contradictory to the result of Huang et al. (30), who found no significant influence of technological anxiety on the usage intention of gerontechnology by seniors in Beijing, China. The discrepancy might be due to the differences in the education level of the participants between their study and ours. In their study, nearly half of the older adults had an education level of university and above, while in our study, the majority of participants had an education level of high school and below (about 70%). Previous research on the digital divide also found that individuals with a higher education level had relatively less fear of new technologies and were more willing to use them (65). Therefore, our results suggested that more attention needed to be given to addressing technology anxiety among older adults with lower levels of education, which could be a significant obstacle to OMS acceptance.

In our study, trust (Tru) was found to be a positively influential determinant of the usage intention of OMS older adults, which confirmed the findings of previous studies (14, 38). Trust reflects the faith of older adults in the professionalism and reliability of doctors and OMS platforms, which was an important driver for the adoption of OMS among older adults. Especially in China, the increasing number of medical disputes in recent years (66), and the lifetime threat of the effects of misdiagnosis and improper treatment (41) all suggest that OMS providers need to convey the credibility of their services and guarantee service quality to their users.

Besides, perceived risk (PR) was found to be another significant barrier to the acceptance of OMS among older adults. This finding of our study was aligned with the results of previous studies (14). The risk issue is a key factor that has long influenced older adults to use Internet-related technologies and services since the rise of the Internet era (37, 67). For OMS, user information includes not only personal identification and financial information but also sensitive health information and privacy. If older adults feel that the security of their information is not fully guaranteed, it will reduce their willingness to accept and adopt OMS.

### 6.2 The influences of heterogeneity among older adults on the usage intention of OMS

In this study, three out of the four moderating variables showed significant influence on the relationship between the main constructs and usage intention. First, age differences among the participants were found to significantly moderate the effects of EE, FC, and TA on UI. That was, there were stronger effects of EE, FC, and TA on the usage intention of OMS for older adults aged 75 and above. Our results were inconsistent with the findings of previous studies on the middle-aged and young population, which did not find a significant effect of age on the usage intention of OMS (13). Our findings might reflect the specificity of the older population, possibly because older adults with advanced age were experiencing a more severe decline in learning and memory abilities, and they might learn a new technology slower and were more prone to forgetting it (68). Meanwhile, their basic devices were relatively old (such as mobile phones, computers, and tablets that might be the old ones used by their children), and most of them had less time and experience with smartphones, computers, and the internet (69). All of the above made them more afraid and anxious about using OMS, and they placed greater emphasis on ease of use. They also believed that more support should be provided for them to learn how to use OMS.

Second, the education level of the participants revealed significant differences in the moderating effects on the relationship between EE and UI and between TA and UI, with stronger effects for the having education experience of secondary school or below. These results were in line with the findings of many previous studies, which also showed that older adults with lower education levels had poorer technological adaptability (25, 70). The possible reasons for our results were compared with other older adults, their low educational level makes it more difficult for them to understand and learn about OMS. Moreover, they are more worried about making operational mistakes and are more likely to experience choice anxiety when faced with a large amount of information on OMS. Our results also suggested that special attention should be paid to older adults with low educational levels in the process of filling these “digital divides” (71).

Lastly, the differences in the health status of the participants posed significantly different influences on the relationship between Tru and UI and between PV and UI. Previous studies also found that the health status of older adults significantly influenced their adoption of health technologies (19, 25), which was similar to the results of this study. For older adults with poor health, trust had a stronger effect on the usage intention of OMS. This result might be because their diseases were complex and their trust in the professionalism and service quality of OMS determined whether they adopt it or not. Some participants with poor health were also skeptical about the role of OMS, believing that it might not be able to solve their serious and complex diseases due to the limitations of no face-to-face contact (18). Furthermore, older adults with poor health often suffered from multiple diseases and chronic illnesses, so they required medical treatment and consultation more frequently, which also led to an increase in the costs and economic burden. Therefore, they are more sensitive and value the price of OMS, compared with other older adults.

### 6.3 Contributions to theory

This study has significant theoretical contributions to the research on the adoption of OMS. Our research focused on older adults, an under-researched demographic in existing literature, and comprehensively considered the factors closely associated with the psychology of this special population, including technological anxiety, trust, price value, and perceived risk. Additionally, it analyzed the influence of individual characteristics such as age, gender, educational level, and health status, rendering the study uniquely applicable to the context of OMS. Consequently, this study enriches the existing literature on OMS, an emerging technological service, in the context of China, a country grappling with severe population aging, unevenly distributed healthcare resources, and high medical costs.

This study also makes innovative contributions to UTAUT2. It enriches the utilization of this theoretical model in the limited research on technology acceptance among older adults. It is a forefront research that uniquely extends the application field of UTAUT2 to the OMS field as to date, empirically validates its availability and adaptability in a rather under-researched setting. Besides, our research advances the understanding of price value, an important variable in consumption contexts, which is less commonly used in the existing UTAUT2 literature.

### 6.4 Implications for practice

In addition to theoretical contributions, some recommendations are also provided to promote the adoption of OMS among older adults based on our findings. First, service providers should optimize the functions of the service system, such as providing user customization to meet the diverse medical needs of older adults, and they should also highlight the advantages of OMS such as the convenience of breaking through the limitations of time and space. Meanwhile, they need to set reasonable prices and provide discounts or a freemium business model (12) to reduce costs, and improve service quality to enhance user’s endorsement of the service value.

Second, software developers should improve the interface design, operation process, and interaction methods of the software to enhance its learnability and accessibility (68). For example, it is necessary to set up simpler interface layouts, simpler operation processes, and more natural ways of information input and acquisition, such as using voice interaction and face recognition. They also need to enhance the security of the system by protecting user information and privacy such as using anonymous or steganography design, and setting reversible operations during and after payment.

Third, governments and communities should take advantage of the social network and preferred information channels of older adults such as newspapers, television, and offline gatherings to promote OMS and provide knowledge guidance. Moreover, it is necessary to encourage older adults to learn to use OMS more to dispel their fear and anxiety of using OMS, especially for older adults with advanced age and low educational levels. For example, communities can organize older adults to participate in training programs on using OMS.

Finally, it is necessary to establish a real-name registration system and authoritative authentication. Administrative departments should strengthen their supervision and require the doctors and pharmacists who provide services to comply with industry norms and ethics, to improve service quality and explain the limitations of OMS to patients (3).

## 7 Conclusion

Online medical services have great market potential and rich benefits for older adults. This study proposed a modified UTAUT2 model integrating new constructs namely technology anxiety, trust, and perceived risk to adapt to the context of adopting OMS by older adults. Our empirical research found that performance expectancy, effort expectancy, social influence, and trust were the drivers influencing the adoption of OMS, while technology anxiety and perceived risk were the barriers hindering the adoption of OMS. Facilitating conditions and price value did not show a significant influence on usage intention of OMS in general, while facilitating conditions had a significant effect on usage intention for older adults with advanced age, and price value had a significant effect on usage intention for older adults with poor health. Age, education level, and health status moderated the relationship between the main constructs and usage intention significantly. Service providers, policymakers, market practitioners, governments, and administrative departments need to pay attention to the influence of these drivers and barriers on the adoption of OMS by older adults, and fully consider the heterogeneity of this special population. We need to ensure the successful implementation of OMS among older adults by offering high-quality service designs, formulating effective strategies, and developing better promotion approaches.

### 7.1 Limitations and future research directions

This study also had some limitations. First, the sample of this study was limited to a typical city in eastern China. The adoption of OMS is also influenced by regional economic level, medical infrastructure, and policy environment. Inter-regional disparities (such as comparison with central/western China) may affect the heterogeneity of the conclusions. Therefore, caution is required when generalizing these findings. Future studies should broaden the sample coverage and thereby test and validate the findings through cross-regional and cross-cultural studies. Second, this study did not find a significant influence of gender difference on usage intention. It was inconsistent with previous research which found a gender gap existed in technology acceptance. Further research is needed to re-examine and further investigate the essential role of gender. Third, this study employed a cross-sectional survey design, which only provided observations at a single time node and included exclusively non-users of OMS. Longitudinal research is warranted to explore changes in older adults’ perceptions of OMS and their psychological states following actual service use. Such studies would also enable the investigation of the real-world evaluation of price value and the influences of experiences and habits. Finally, the cognitive and psychological processes involved in older adults’ transition from awareness to adoption of new technologies are complex. These intricate journeys may be further shaped by some external factors and mediating variables such as technology readiness, social support, self-efficacy, and health literacy, which also need to be explored in future research.

## Data Availability

The original contributions presented in the study are included in the article/Supplementary material, further inquiries can be directed to the corresponding author.
