# Supplementary material for: Exploring the factors influencing the adoption of online medical services by older adults: a modified UTAUT2 based study
Source: Front Public Health. 2025 Apr 3;13:1559701. doi: 10.3389/fpubh.2025.1559701 (PMC12003354; doi:10.3389/fpubh.2025.1559701)
Supplement: Supplementary file 1 [file Data_Sheet_1.doc]

**Appendix A: Measurement Constructs and Items of This Study**

| Constructs | Items | Description | References |
| --- | --- | --- | --- |
| Performance expectancy (PE) | PE1 | I find online medical services useful in my daily life. | [14, 27] |
|  | PE2 | Using online medical services helps me access to healthcare services more conveniently. |
|  | PE3 | Using online medical services improves the quality of my health care. |
| Effort expectancy (EE) | EE1 | Learning how to use online medical services is easy for me. | [27] |
|  | EE2 | My interaction with the platform of online medical services is clear and understandable. |
|  | EE3 | I find online medical services easy to use. |
|  | EE4 | It is easy for me to become skillful at using online medical services. |
| Social influence (SI) | SI1 | People who are important to me think I should use online medical services. | [27] |
|  | SI2 | People whose opinions that I value prefer that I use online medical services. |
|  | SI3 | People around me who have experience of using online medical services will affect my use of online medical services. |
| Facilitating conditions (FC) | FC1 | I have sufficient conditions/equipments such as smartphone and stable network to use online medical services. | [13, 27] |
|  | FC2 | I have sufficient knowledge for using online medical services. |
|  | FC3 | I can get help and guidance when I use online medical services. |
|  | FC4 | Online medical services are compatible with other technologies I use. |
| Technological anxiety (TA) | TA1 | Using online medical services makes me feel nervous. | [25, 31] |
|  | TA2 | I feel apprehensive about using online medical services. |
|  | TA3 | I hesitate to use online medical services for fear of making mistakes I can not correct. |
| Trust (Tru) | Tru1 | Online medical services are trustworthy for improving my health care. | [14, 53] |
|  | Tru2 | I could trust the authenticity and reliability of online medical services. |
|  | Tru3 | I feel confident with the professionalism of doctors and pharmacists when I use online medical services. |
| Price value (PV) | PV1 | It enables me to use online medical services at a reasonable price. | [27] |
|  | PV2 | Online medical services are good value for the money. |
|  | PV3 | At the current price, online medical services provide a good value. |
| Perceived risk (PR) | PR1 | I’m worried about the risk of personal information leaking online | [4, 22] |
|  | PR2 | I’m worried about that there may be financial losses during the process of paying for online medical services. |
|  | PR3 | Using online medical services may lead to privacy violation. |
| Usage intention (UI) | UI1 | I intend to use online medical services in the future. | [13, 27] |
|  | UI2 | I am willing to learn how to use online medical services. |
|  | UI3 | I plan to use online medical services frequently in my daily life. |

**Appendix B: Full Factor Loadings Matrix**

| Items | PE | EE | SI | FC | TA | Tru | PV | PR | UI |
| --- | --- | --- | --- | --- | --- | --- | --- | --- | --- |
| PE1 | **0.857** | 0.167 | 0.093 | 0.212 | 0.048 | 0.115 | 0.033 | 0.085 | 0.024 |
| PE2 | **0.931** | 0.109 | 0.132 | 0.075 | 0.082 | 0.043 | 0.107 | 0.059 | 0.132 |
| PE3 | **0.929** | 0.048 | 0.085 | 0.123 | 0.115 | 0.072 | 0.062 | 0.139 | 0.055 |
| EE1 | 0.135 | **0.834** | 0.228 | 0.168 | 0.036 | 0.063 | 0.145 | 0.041 | 0.076 |
| EE2 | 0.062 | **0.876** | 0.113 | 0.095 | 0.127 | 0.019 | 0.082 | 0.137 | 0.087 |
| EE3 | 0.117 | **0.881** | 0.162 | 0.204 | 0.045 | 0.107 | 0.027 | 0.070 | 0.033 |
| EE4 | 0.089 | **0.840** | 0.195 | 0.147 | 0.069 | 0.142 | 0.054 | 0.098 | 0.021 |
| SI1 | 0.107 | 0.185 | **0.885** | 0.059 | 0.084 | 0.037 | 0.133 | 0.066 | 0.091 |
| SI2 | 0.043 | 0.122 | **0.906** | 0.116 | 0.105 | 0.095 | 0.047 | 0.109 | 0.103 |
| SI3 | 0.155 | 0.063 | **0.946** | 0.081 | 0.027 | 0.078 | 0.101 | 0.115 | 0.084 |
| FC1 | 0.239 | 0.217 | 0.067 | **0.770** | 0.146 | 0.089 | 0.103 | 0.145 | 0.062 |
| FC2 | 0.116 | 0.182 | 0.134 | **0.892** | 0.073 | 0.128 | 0.063 | 0.093 | 0.118 |
| FC3 | 0.095 | 0.169 | 0.112 | **0.852** | 0.090 | 0.053 | 0.142 | 0.071 | 0.086 |
| FC4 | 0.162 | 0.249 | 0.089 | **0.793** | 0.113 | 0.073 | 0.076 | 0.155 | 0.061 |
| TA1 | 0.027 | 0.038 | 0.104 | 0.135 | **0.935** | 0.145 | 0.045 | 0.126 | 0.104 |
| TA2 | 0.113 | 0.095 | 0.139 | 0.079 | **0.869** | 0.111 | 0.091 | 0.058 | 0.082 |
| TA3 | 0.068 | 0.127 | 0.085 | 0.117 | **0.880** | 0.097 | 0.038 | 0.110 | 0.067 |
| Tru1 | 0.142 | 0.053 | 0.047 | 0.068 | 0.096 | **0.858** | 0.132 | 0.112 | 0.104 |
| Tru2 | 0.112 | 0.139 | 0.093 | 0.087 | 0.077 | **0.913** | 0.106 | 0.125 | 0.128 |
| Tru3 | 0.083 | 0.113 | 0.134 | 0.059 | 0.108 | **0.791** | 0.144 | 0.093 | 0.101 |
| PV1 | 0.103 | 0.082 | 0.110 | 0.136 | 0.059 | 0.109 | **0.920** | 0.166 | 0.053 |
| PV2 | 0.068 | 0.109 | 0.197 | 0.088 | 0.094 | 0.134 | **0.867** | 0.102 | 0.107 |
| PV3 | 0.142 | 0.072 | 0.105 | 0.095 | 0.127 | 0.083 | **0.903** | 0.079 | 0.152 |
| PR1 | 0.095 | 0.154 | 0.072 | 0.106 | 0.082 | 0.098 | 0.162 | **0.865** | 0.061 |
| PR2 | 0.139 | 0.088 | 0.129 | 0.121 | 0.104 | 0.140 | 0.107 | **0.963** | 0.094 |
| PR3 | 0.112 | 0.102 | 0.097 | 0.133 | 0.147 | 0.074 | 0.113 | **0.814** | 0.113 |
| UI1 | 0.077 | 0.059 | 0.082 | 0.053 | 0.075 | 0.119 | 0.077 | 0.092 | **0.941** |
| UI2 | 0.103 | 0.114 | 0.077 | 0.072 | 0.122 | 0.149 | 0.082 | 0.131 | **0.856** |
| UI3 | 0.137 | 0.088 | 0.102 | 0.047 | 0.093 | 0.105 | 0.124 | 0.103 | **0.862** |

Note. Factor loadings greater than 0.7 are highlighted.
